# Novel Mechanism of the Pericyte-Myofibroblast Transition in Renal Interstitial Fibrosis: Core Fucosylation Regulation

**DOI:** 10.1038/s41598-017-17193-5

**Published:** 2017-12-05

**Authors:** Nan Wang, Yiyao Deng, Anqi Liu, Nan Shen, Weidong Wang, Xiangning Du, Qingzhu Tang, Shuangxin Li, Zach Odeh, Taihua Wu, Hongli Lin

**Affiliations:** 1 Department of Nephrology, The First Affiliated Hospital of Dalian Medical University, Key Laboratory of Kidney Disease of Liaoning Province, The Center for the Transformation Medicine of Kidney Disease of Liaoning Province, No. 222 Zhongshan Road, Dalian, 116011 China; 2 Graduate School of Dalian Medical University, 9 Western Section, Lvshun South Street, Lvshunkou District, Dalian, 116044 China; 3 International Education College of Dalian Medical University, 9 Western Section, Lvshun South Street, Lvshunkou District, Dalian, 116044 China; 4 Departments of Respiratory Medicine, The First Affiliated Hospital of Dalian Medical University, 222# Zhongshan Road, No. 222 Zhongshan Road, Dalian, 116011 China; 5 Department of Nephrology, Chinese PLA General Hospital, Chinese PLA Institute of Nephrology, State Key Laboratory of Kidney Diseases, National Clinical Research Center for Kidney Diseases, Fuxing Road 28, Haidian District, Beijing, 100853 China

## Abstract

Pericytes have been identified as a major source of myofibroblasts in renal interstitial fibrosis (RIF). The overactivation of several signaling pathways, mainly the TGF-β and PDGF pathways, initiates the pericyte-myofibroblast transition during RIF. Key receptors in these two pathways have been shown to be modified by fucosyltransferase 8 (FUT8), the enzyme that catalyzes core fucosylation. This study postulated that core fucosylation might play an important role in regulating the pericyte transition in RIF. The data showed that core fucosylation increased with the extent of RIF in patients with IgA nephropathy (IgAN). Similarly, core fucosylation of pericytes increased in both a unilateral ureteral occlusion (UUO) mouse model and an *in vitro* model of pericyte transition. Inhibition of core fucosylation by adenoviral-mediated *FUT8* shRNA *in vivo* and *FUT8* siRNA *in vitro* significantly reduced pericyte transition and RIF. In addition, the activation of both the TGF-β/Smad and PDGF/ERK pathways was blocked by core fucosylation inhibition. In conclusion, core fucosylation may regulate the pericyte transition in RIF by modifying both the TGF-β/Smad and PDGF/ERK pathways. Glycosylation might be a novel “hub” target to prevent RIF.

## Introduction

Renal interstitial fibrosis (RIF) is a common final outcome of most progressive chronic kidney diseases (CKDs). A progressive increase in myofibroblasts in the renal interstitial space is a critical cause of fibrosis^1–3^. Recently, pericytes have been identified as an important source of myofibroblasts during RIF^4–7^. Pericytes are perivascular cells attached to the abluminal surface of capillaries, and share developmental origins with fibroblasts. Under physiological conditions, pericytes stabilize vascular walls and maintain vascular quiescence and vascular integrity. Under pathological conditions, pericytes are activated, detach from vascular walls, and transition to myofibroblasts^8–13^. Meanwhile, the loss of pericytes within the perivascular compartment results in vulnerable capillaries, which are prone to instability, pathological angiogenesis, and ultimately rarefaction^14^.

The overactivation of several signaling pathways has been shown to be significantly involved in the pericyte-myofibroblast transition. In addition to the well-known classical TGF-β signaling pathways, the PDGF pathway has also been confirmed to induce the proliferation and differentiation of pericytes into myofibroblasts^15–19^. However, an appropriate method or target that simultaneously prevents the overactivation of both pathways is not available.

Based on the results of our previous study and other reports, core fucosylation modifies TGF-βR and PDGFRβ; furthermore, inhibition of the core fucosylation of TGF-βR alleviates RIF in rat models of unilateral ureteral obstruction (UUO) and renal tubular cell injury *in vitro* ^20,21^. Core fucosylation of proteins is catalyzed by α1,6-fucosyltransferase (FUT8) in the Golgi apparatus, which adds fucose to the innermost GlcNAc residue of N-linked oligosaccharides on glycoproteins, and this modification is preferentially recognized by *Lens culinaris* lectin (LCA)^22–24^.

This study postulated that core fucosylation might be a potential target to simultaneously prevent these two receptors from triggering the activation of downstream intermediates. We investigated the effect of core fucosylation on the activities of both the TGF-β/Smad and PDGF/ERK pathways during the pericyte transition in RIF. The down-regulation of core fucosylation prevented the pericyte transition and RIF by inhibiting the activities of both the TGF-β/Smad and PDGF/ERK pathways. This study is the first to suggest that the core fucosylation of pericytes represents a promising “hub” target for RIF.

## Results

### Core Fucosylation of Pericytes is Increased in Renal Biopsies of Patients with IgAN

**Figure 1:**
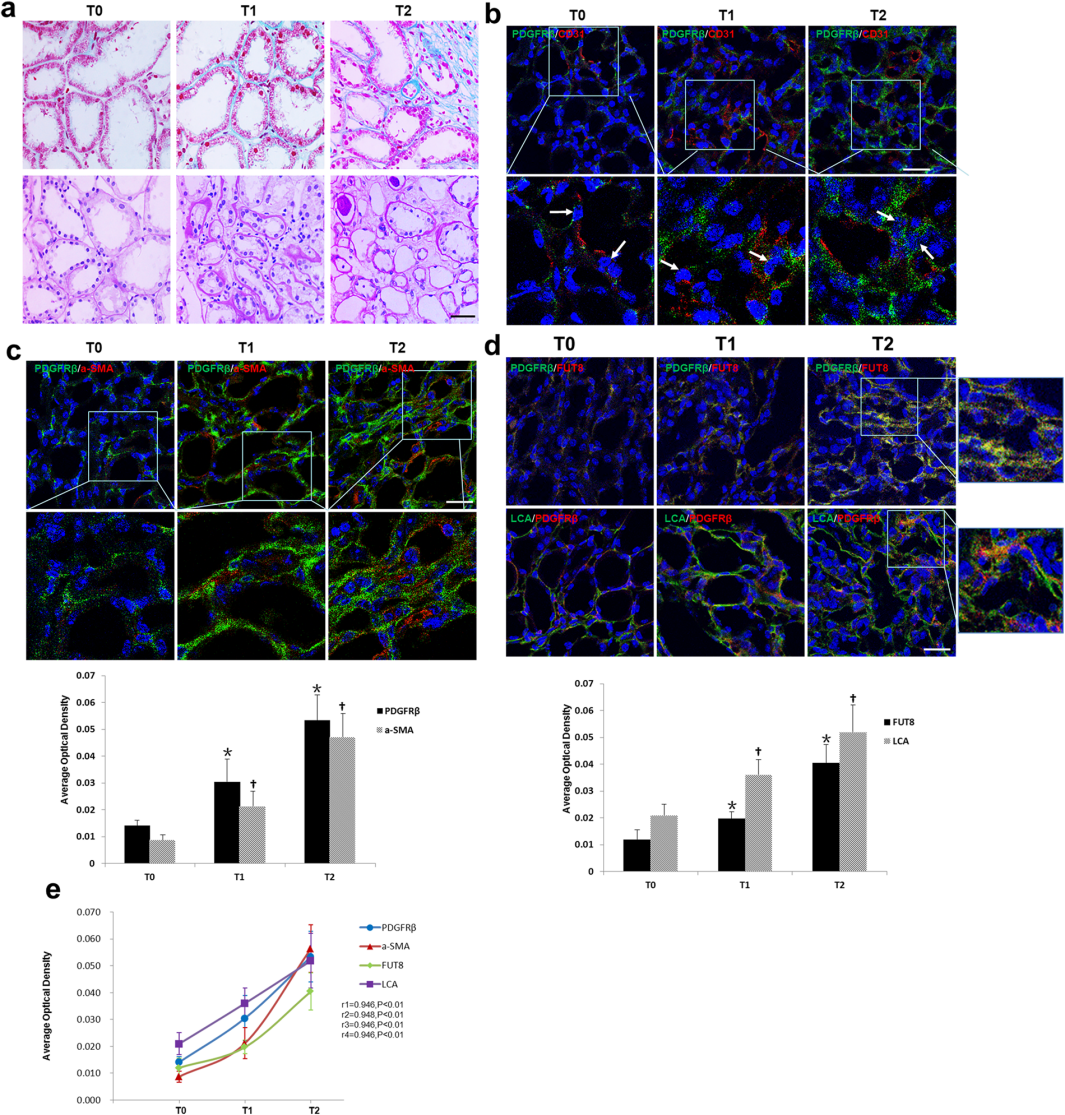
The pericyte-myofibroblast transition and core fucosylation were increased in patients with different Oxford classifications of IgAN. (**a**) Representative images of Masson’s trichrome-stained and PAS-stained sections from patients with different Oxford T classifications of IgAN. (**b**) Representative images of PDGFRβ (green) and CD31 (red) staining; white arrows indicate pericytes. Quantification is shown in the lower panel. (**c**) Representative images of PDGFRβ (green) and α-SMA (red) staining. Quantification is shown in the lower panel. (**d**) Representative images of dual staining for PDGFRβ (green) and FUT8 (red) and dual staining for LCA (green) and PDGFRβ (red). Quantification is shown in the lower panel. (**e**) Spearman’s correlation coefficients were used to examine the differences in the fluorescence intensity of immunostaining for PDGFRβ, α-SMA, FUT8, and LCA in patients with different Oxford T classifications of IgAN. r, Spearman’s correlation coefficient; r1, PDGFRβ; r2, α-SMA; r3, FUT8; r4, LCA. Scale bar, 50 μm. *P < 0.01, ^†^ *P* < 0.01. * and ^†^ indicate the comparison of the T1 or T2 group with the T0 group.

Thirty-two patients with IgAN were divided into three groups based on T scores of renal biopsies according the Oxford IgAN classification^22–24^. Clinical characteristics of the patients are shown in Table 1. Serum creatinine levels were significantly increased, whereas the estimated glomerular filtration rate (eGFR) was decreased in the T2 patients compared to patients in the T0 group (*P* < 0.05). A greater number of pericytes (PDGFRβ+) detached from interstitial endothelial cells (CD31+) in patients with T1 and T2 scores (Fig. 1a and b), and PDGFRβ+ cells showed enhanced expression of α-SMA (Fig. 1c). The LCA and FUT8 levels in pericytes increased as the T scores increased (Fig. 1d). According to the correlation analysis, the detachment, transition, and core fucosylation of pericytes were significantly related to T scores (Fig. 1e, r1 = 0.946, r2 = 0.948, r3 = 0.946, r4 = 0.9, *P* < 0.01). These findings indicated that core fucosylation of pericytes increased when pericytes were activated in the RIF process of patients with IgAN.

**Table 1:** Clinical characteristics of the 32 IgAN patients.

Clinical Characteristics	T0 (n = 10)	T1 (n = 13)	T2 (n = 9)	P value
Age, median (IQR) (year)	41 (27–51)	38 (31–48)	36 (34–53)	P_1_ = 0.477, P_2_ = 0.243
Sex, male (%)	5 (50)	5 (38.5)	4 (44.4)	—
IgAN Duration (month)	18.3 ± 12.1	26.8 ± 27.8	22.4 ± 17.4	P_1_ = 0.19, P_2_ = 0.275
Systolic BP (mmHg)	122.3 ± 10	125.9 ± 15.5	131 ± 13.3	P_1_ = 0.264, P_2_ = 0.062
Diastolic BP (mmHg)	79.8 ± 6.7	79.8 ± 9.8	85.3 ± 12.5	P_1_ = 0.495, P_2_ = 0.119
Mean arterial BP (mmHg)	94 ± 6.5	95.2 ± 10.9	100.6 ± 12	P_1_ = 0.377, P_2_ = 0.074
Twenty-four hour urinary protein (mg/24 h)	621.3 ± 646.9	614 ± 386.6	1634.5 ± 890.9	P_1_ = 0.487, P_2_ = 0.005*
Albumin (g/l)	42.1 ± 3	39.5 ± 4.8	39.2 ± 4	P_1_ = 0.077, P_2_ = 0.04*
Serum creatinine (umol/l)	64.7 ± 12.9	71.8 ± 28.6	90.2 ± 32.8	P_1_ = 0.239, P_2_ = 0.018*
Urine pH	6.35 ± 0.7	6 ± 0.8	5.6 ± 0.4	P_1_ = 0.176, P_2_ = 0.006*
^eGFR^MDRD (ml/min per 1.73 m^2^)	115.7 ± 23.3	108.9 ± 39.4	85 ± 35.5	P_1_ = 0.316, P_2_ = 0.018*
Urine specific gravity	1.018 ± 0.008	1.019 ± 0.008	1.017 ± 0.005	P_1_ = 0.371, P_2_ = 0.411

*P*_1_, T1 vs T0, *P*_2_, T2 vs T0, *P < *0.05*.

### Core Fucosylation of Pericytes is Increased in UUO Mouse Models

**Figure 2:**
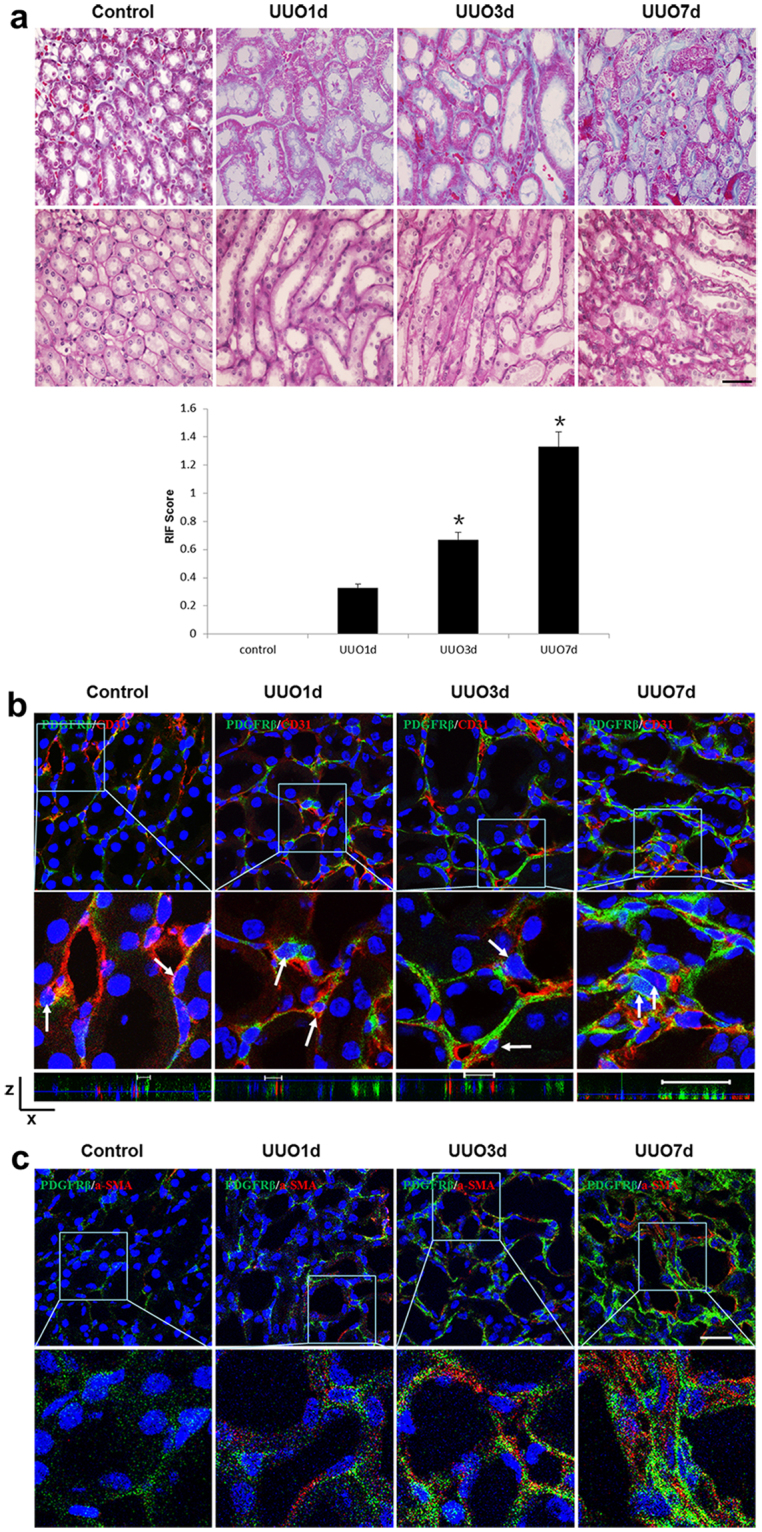
The pericyte-myofibroblast transition and core fucosylation were increased in UUO mouse models (n = 5). (**a**) Representative images of Masson’s trichrome-stained and PAS-stained UUO kidney sections. (**b**) Representative images of PDGFRβ (green) and CD31 (red) staining; the high-magnification image shows that pericytes had detached from endothelial cells; white arrows indicate pericytes. A 3D image reconstruction was performed to show the detachment of the pericytes. (**c**) Representative images of PDGFRβ (green) and α-SMA (red) staining. Scale bar, 50 μm. *P < 0.01. *Indicate the comparison of the control group with the UUO group.

**Figure 3:**
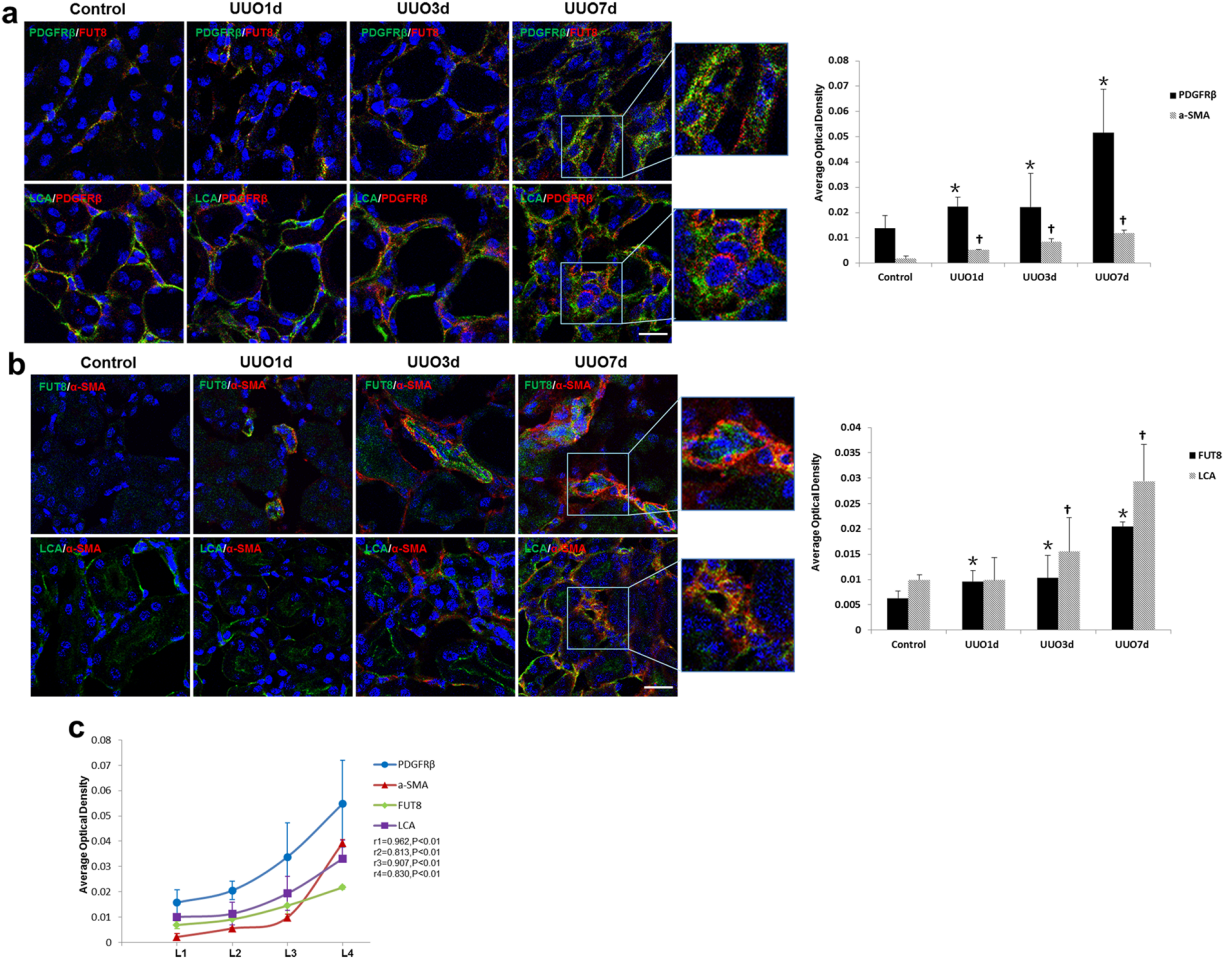
The pericyte-myofibroblast transition and core fucosylation were increased in UUO mouse models (n = 5). (**a**) Representative images of dual staining for PDGFRβ (green) and FUT8 (red) and dual staining for LCA (green) and PDGFRβ (red). (**b**) Representative images of dual staining for FUT8 (green) and α-SMA (red) and dual staining for LCA (green) and α-SMA (red). (**c**) Spearman’s correlation coefficients were used to examine the differences in the fluorescence intensity of immunostaining for PDGFRβ, α-SMA, FUT8, and LCA in mice with different RIF scores. r, Spearman’s correlation coefficient; r1, PDGFRβ; r2, α-SMA; r3, FUT8; r4, LCA. Scale bar, 50 μm. *P < 0.01, ^†^ *P* < 0.01. * and ^†^ indicate the comparison of the control group with the UUO group.

We further detected the pericyte transition and core fucosylation in UUO mouse models (Fig. 2a). Pericytes began to detach from the interstitial endothelial cells as early as 1 day after UUO. A greater number pericytes subsequently detached, and the expression of α-SMA increased in renal interstitial areas over time (Fig. 2b and c). Immunofluorescence co-staining was used to evaluate the relationship between core fucosylation, pericytes and myofibroblasts. The FUT8 and LCA levels in pericytes were substantially increased in a time-dependent manner, and similar increases were also observed in myofibroblasts (Fig. 3a and b). According to the correlation analysis, the detachment, transition, and core fucosylation of pericytes were significantly related to the severity of RIF (Fig. 3c, r1 = 0.962, r2 = 0.813, r3 = 0.907, r4 = 0.830, *P* < 0.01).

### Down-regulation of Core Fucosylation Alleviates the Pericyte Transition *In Vitro*

**Figure 4:**
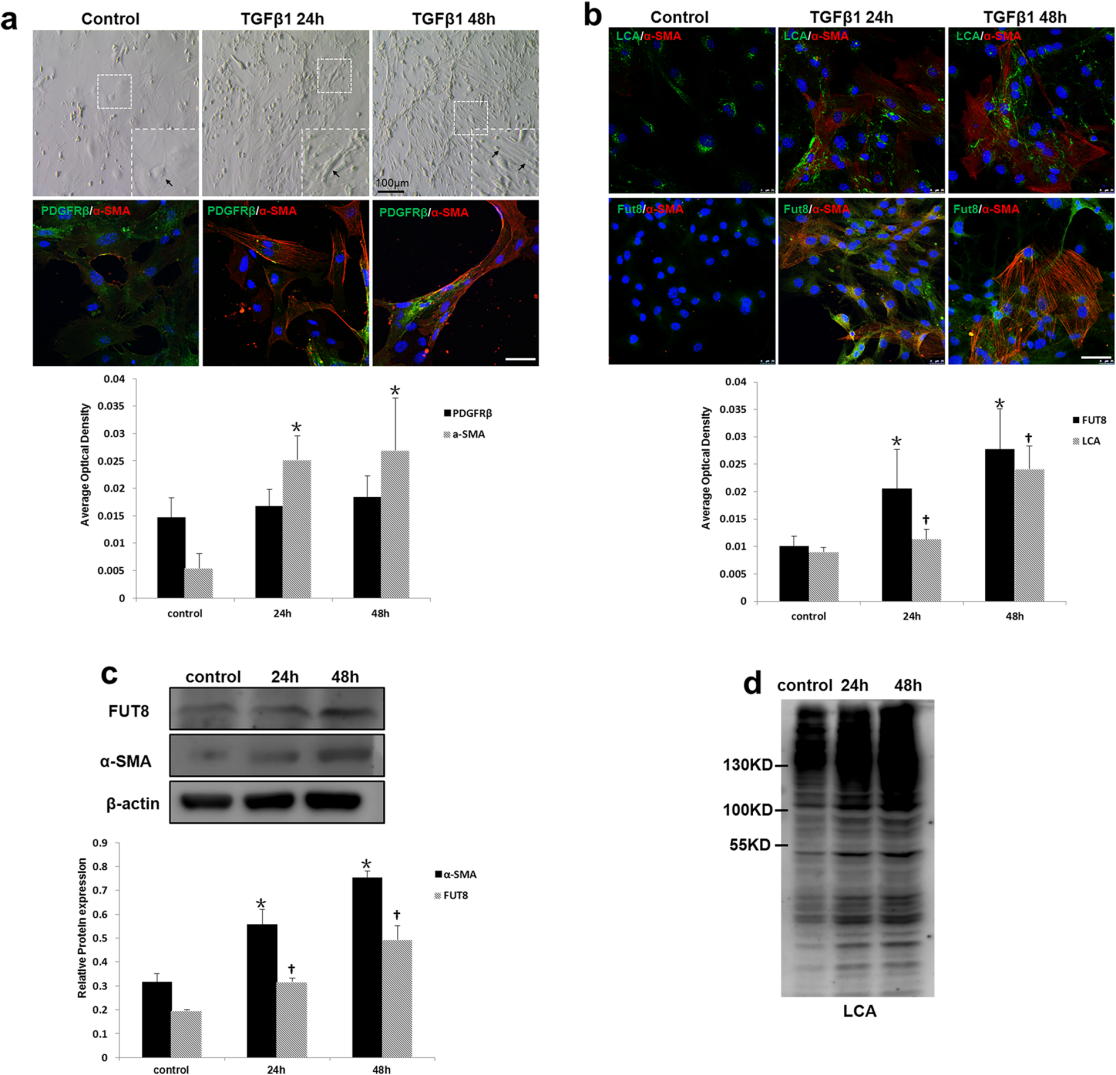
Core fucosylation was increased during the TGFβ1-induced pericyte-myofibroblast transition *in vitro*. Primary cultures of pericytes were incubated with TGFβ1 (10 ng/ml) for 24 or 48 h. (**a**) Representative bright-field images of morphological alterations in pericytes, where black arrows indicate pericytes, and representative images of PDGFRβ (green) and α-SMA (red) staining are shown. (**b**) Representative images of dual staining for FUT8 (green) and α-SMA (red) and dual staining for LCA (green) and α-SMA (red) staining. (c) PDGFRβ, α-SMA, and FUT8 levels were assessed using Western blot analyses. (**d**) Lectin blot analyses. Quantification is shown in the lower panel. Scale bar, 50 μm. *P < 0.01, ^†^ *P* < 0.01. * and ^†^indicate the comparison of the control group with the TGFβ1 group; ^#^ indicates the comparison of the FUT8 knockdown group with the TGFβ1 group.

**Figure 5:**
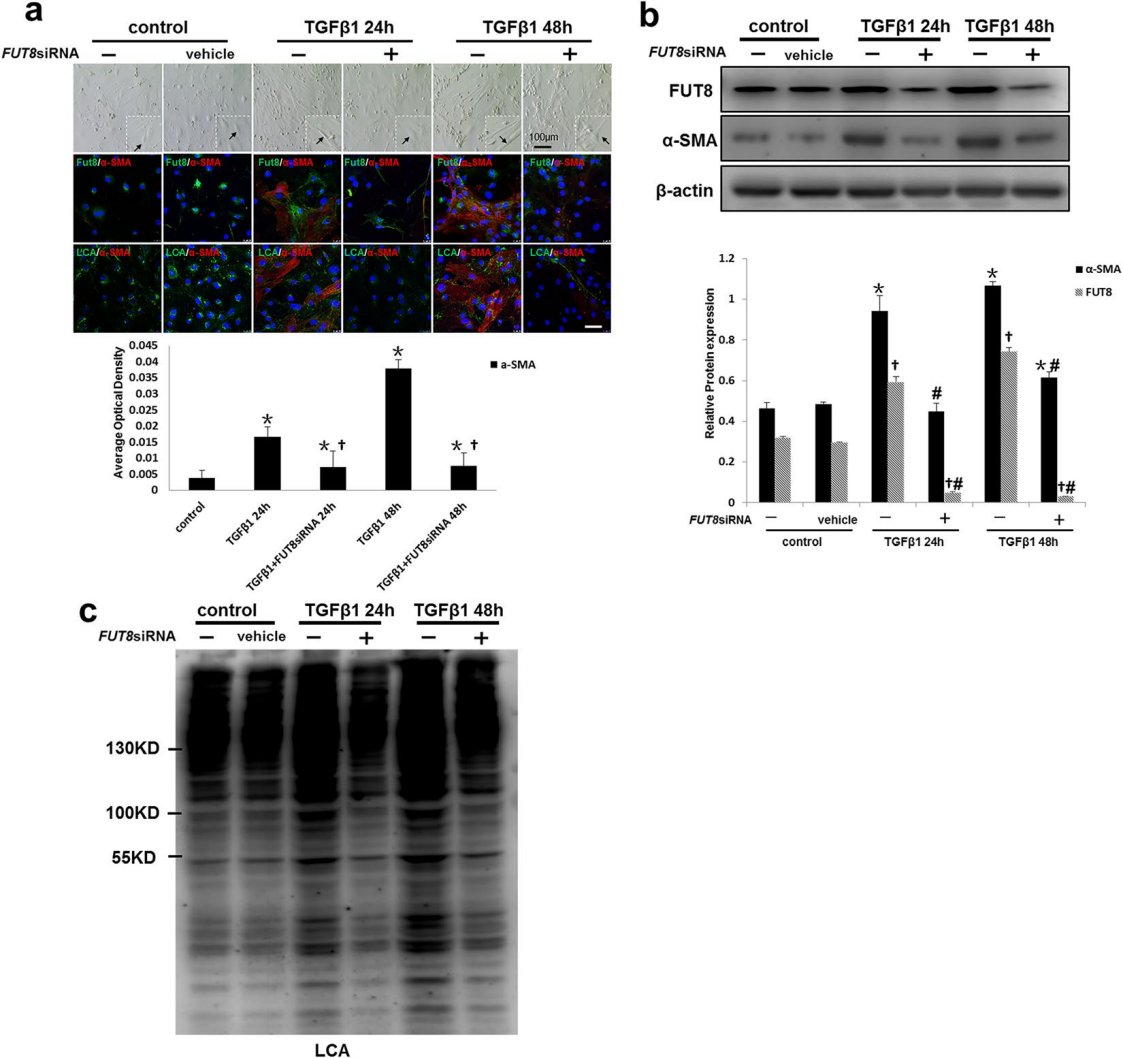
Core fucosylation was down-regulated and the pericyte-myofibroblast transition was inhibited upon FUT8 knockdown *in vitro*. Primary cultures of pericytes were incubated with TGFβ1 (10 ng/ml) for 24 or 48 h. (**a**) Representative bright-field images of morphological changes in pericytes, where black arrows indicate pericytes, and representative images of FUT8 (green), LCA (green), and α-SMA (red) staining are shown. Quantification is shown in the lower panel. (**b**) FUT8 and α-SMA levels were assessed using Western blot analyses. Quantification is shown in the lower panel. (**c**) Lectin blot analyses. Cell lysates were subjected to a lectin blot analysis using LCA. Representative data are shown. Quantification is shown in the lower panel. Scale bar, 50 μm. *P < 0.01, ^†^ *P* < 0.01. * and ^†^ indicate the comparison of the control group with the TGFβ1 group; # indicates the comparison of the FUT8 knockdown group with the TGFβ1 group.

We then established an *in vitro* pericyte transition model to further investigate the role of core fucosylation. C57BL/6 mouse kidney pericytes were isolated and cultured *in vitro* (Supplementary Fig. S1A–C). The profibrotic factor TGF-β1 was used to induce the pericyte transition. After 24 h of TGF-β1 induction, myofibroblast-like morphological changes were observed in pericytes, along with increased expression of α-SMA (Fig. 4a). Both immunofluorescence staining and Western blot analyses showed that FUT8 and LCA expression increased when pericytes transitioned to myofibroblasts after TGF-β1 induction in a time-dependent manner (Fig. 4b–d). Furthermore an *FUT8* siRNA was used to successfully knockdown FUT8 expression (Supplementary Fig. S2A), and LCA was expressed at very low levels after FUT8 knockdown (Fig. 5c). Myofibroblast-like morphological changes in pericytes were substantially alleviated after FUT8 knockdown, along with a decrease in α-SMA expression (Fig. 5a and b).

### Inhibition of Core Fucosylation Prevents the Pericyte Transition and RIF in UUO Mouse Models

**Figure 6:**
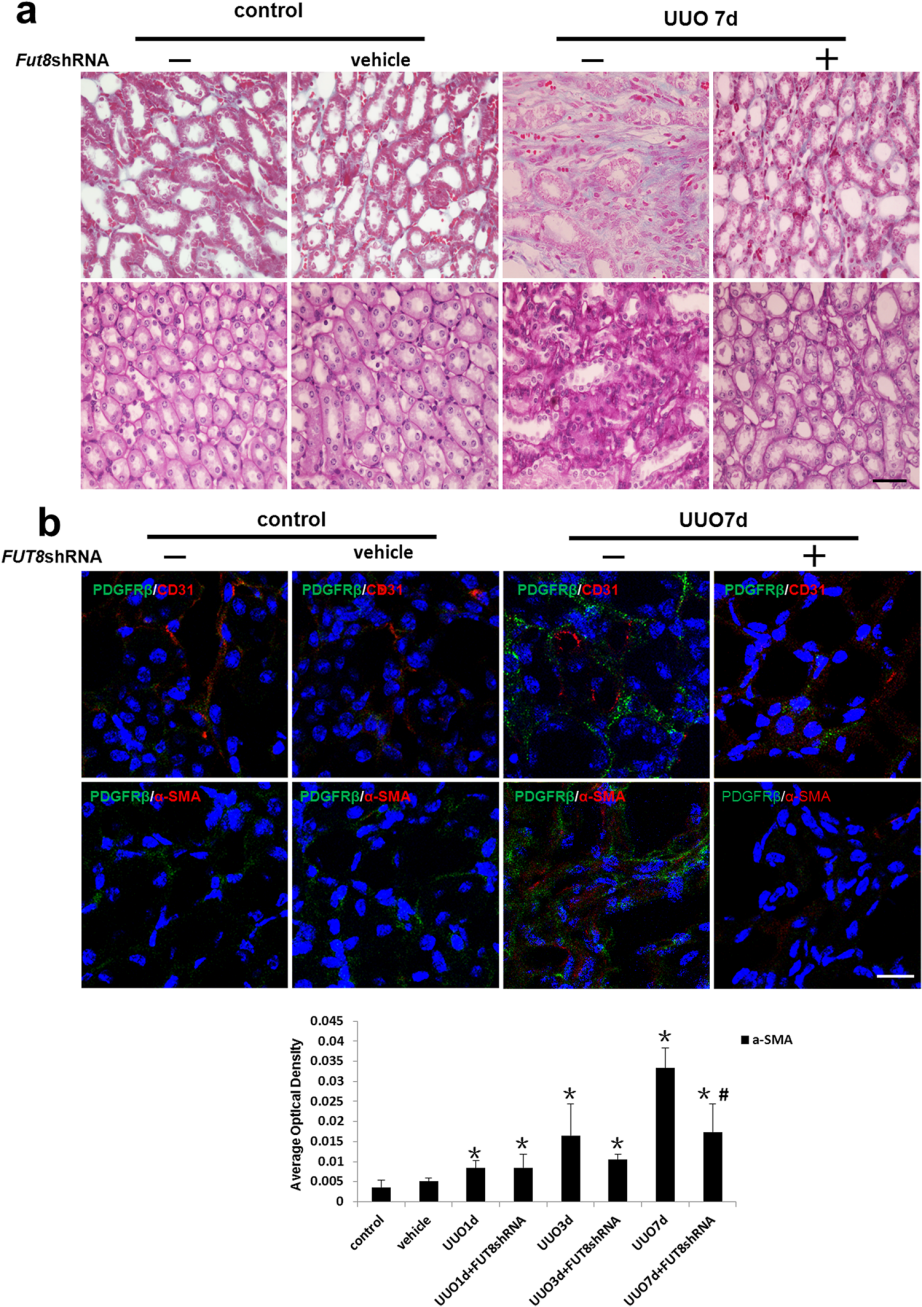
The pericyte-myofibroblast transition was inhibited and RIF was reduced upon FUT8 knockdown *in vivo*. UUO models were established (n = 5). A recombinant adenovirus carrying the *FUT8* shRNA was used to knock down FUT8 expression *in vivo*. (**a**) Representative images of Masson’s trichrome-stained and PAS-stained UUO kidney sections. (**b**) Representative images of dual staining for PDGFRβ (green) and α-SMA (red) staining and dual staining for PDGFRβ (green) and CD31 (red). Quantification of α-SMA staining is shown in the lower panel. Scale bar, 50 μm. *P < 0.01, ^#^ *P* < 0.01. *Indicates the comparison of the control group with the UUO group; ^#^indicates the comparison of the FUT8 knockdown group with the UUO group.

To further confirm the role of core fucosylation *in vivo*, we knocked down FUT8 to evaluate the subsequent pericyte transition and RIF. The *FUT8* shRNA recombinant adenovirus vector used for FUT8 knockdown *in vivo* was constructed as described in our previous research^25^ (Supplementary Fig. S2B and C). Injection with the *FUT8* shRNA recombinant adenovirus dramatically decreased pericyte detachment and the transition to myofibroblasts in renal interstitial areas, followed by the alleviation of RIF in UUO mice (Fig. 6a and b). Based on these findings, core fucosylation played a key regulatory role in the pericyte transition and RIF.

### Core Fucosylation Regulates the Activities of Both the TGF-β/Smad and PDGF/ERK Pathways in the Pericyte Transition

**Figure 7:**
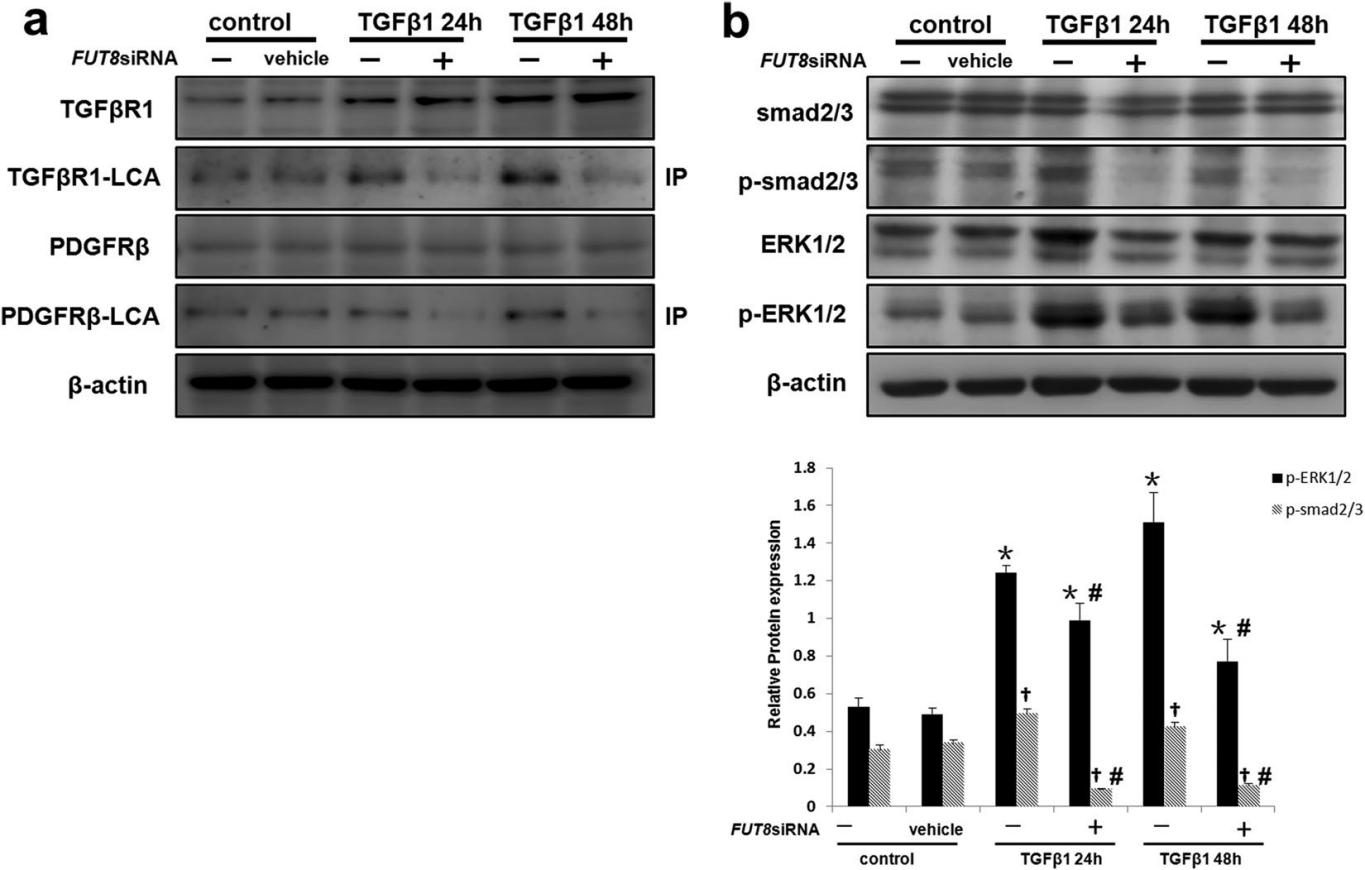
Core fucosylation regulates the pericyte-myofibroblast transition through both the TGFβ/Smad2/3 and PDGF/ERK1/2 pathways *in vitro*. (**a**) TGFβR1 and PDGFRβ levels in total cell lysates were assessed using Western blot analyses. Lectin blot analysis of the immunoprecipitated TGFβR1 and PDGFRβ proteins. TGFβR1 and PDGFRβ were immunoprecipitated from whole cell lysates with anti-TGFβR1 and anti-PDGFRβ antibodies, respectively. The blots were probed with LCA. Representative data are shown. Quantification is shown in the lower panel. (**b**) Smad2/3, p-Smad2/3, ERK1/2, and p-ERK1/2 levels were assessed using Western blot analyses. Total cell lysates were subjected to immunoblotting. Representative data are shown. Quantification is shown in the lower panel. *P < 0.01, ^#^ *P* < 0.01. * and ^†^ indicate the comparison of the control group with the TGFβ1 group; ^#^ indicates the comparison of the FUT8 knockdown group with the TGFβ1 group.

We postulated that the beneficial effects of the *FUT8* siRNA on RIF are due to its simultaneous inactivation of several profibrotic pathways. As TGF-β and PDGF pathways have been recognized as key and classical profibrotic signaling pathways in RIF^15–17^, we further investigated the effect of the *FUT8* siRNA on these pathways. Immunoprecipitation of LCA showed that core fucosylation of both TGF-βR1 and PDGFRβ significantly increased after TGF-β1 induction over time and was alleviated by FUT8 knockdown *in vitro* (Fig. 7a). Moreover, the levels of phosphorylated Smad2/3 and ERK1/2 were significantly decreased in response to FUT8 knockdown (Fig. 7b). Thus, the inhibition of core fucosylation simultaneously inactivated the TGF-β/Smad and PDGF/ERK pathways and alleviated the pericyte transition.

## Discussion

Recent studies have confirmed that pericytes are activated and transition to myofibroblasts, which is the direct cause of RIF, but the regulatory mechanisms are unclear. Most proteins are glycoproteins, and glycosylation has a role in protein function. Our study elucidated the regulatory mechanisms involving glycosylation. Previous reports have shown that FUT8 can globally modify surface antigens, receptors, and adhesion molecules^26–31^. TGF-βR and PDGFRβ have been reported to be modified by core fucosylation, which is directly associated with their function^20,32,33^. Our previous work showed that inhibition of core fucosylation could alleviate RIF in UUO rat models^20,21^. Thus, we hypothesized that core fucosylation modification of multiple signaling pathways might be involved in pericyte transition in RIF.

IgAN is one of the most common causes of CKD, and end-stage renal disease^34^. The kidney prognosis of IgAN is more closely associated with the severity of interstitial injury and fibrosis than that of glomerular lesions^35–37^. In renal biopsies from patients with IgAN, we observed a significant increase in the core fucosylation of pericytes that was highly correlated with the severities of renal interstitial injuries represented by T grading. To the best of our knowledge, we are the first to report this core fucosylation disorder of pericytes in patients with IgAN.

Although pericytes do not have specific markers, the diverse characteristics of pericytes indicate that they have large differentiation capacities. Other studies have found that pericytes lack expression of NG2 but express PDGFR-β in adult mouse kidney^7,38^. Fluorescence-activated cell sorting (FACS) is traditionally applied for PDGFRβ+ pericyte isolation from kidney^16^. However, our preliminary experiment found that the positive isolation percentage was greatly affected by the impurities during tissue preparation, such as lipids or dead cell debris. To minimize the interference of impurities, Percoll was applied to form a gradient density to remove impurities. FACS or magnetic-activated cell sorting (MACS) was then used for pericyte isolation, but MACS increased cell viability.

Consistent with previous reports^6,9,12,39^, we found that pericytes detached from endothelial cells and transitioned to myofibroblasts in primary cultured pericyte transition models and UUO mouse models. In addition, we found that core fucosylation was increased in experimental models and in patients with IgAN. However, core fucosylation did not increase in all pericytes. As shown in the study by Ching-Fang Wu *et al*., pericyte proliferation is substantially increased in the kidneys of subjects with UUO, and our results were consistent with their findings^17^. Thus, we postulate that some pericytes were proliferating and had not completely transitioned to myofibroblasts; thus, core fucosylation levels may start to increase when pericytes are in a pre-transition or transitioned status. As shown in Fig. 3b, almost all myofibroblasts showed high levels of fucosylation. Then, we inhibited FUT8 expression *in vivo* and *in vitro* and found that both the pericyte transition and RIF were dramatically alleviated. These findings indicated that core fucosylation was closely associated with the pericyte transition and RIF.

Pathological activation of pericytes is caused by various factors. The balance of the renal interstitial micro-environment is precisely regulated by elaborate adjacent structures between tubules, capillaries, and cross-talk among cells. Once the micro-environment balance is disrupted, pericyte activation and transition are initiated. Cross-talk, such as among TGF-β, PDGF, VEGF, CTGF, and Wnt, is commonly found among epithelial cells, endothelial cells, and pericytes, as well as within cells. Blockade of a single signaling pathway can inhibit RIF to some extent, but activation of other pathways would increase in compensation^40,41^. TGF-β1 signaling induces the phosphorylation of Smad proteins, which control the expression levels of fibrogenic genes through TGF-βRI and TGF-βRII receptor complexes^25,42^. MAPK signaling pathways in pericytes have a critical function in the progression of renal fibrosis^16,43^. In UUO mouse models, PDGF was reported to stimulate ERK1/2 activation in pericytes^44^. Both the TGF-β/Smad and PDGF/ERK pathways were activated following TGF-β1 induction in the present study, and decreased core fucosylation of receptors reduced the activities of downstream pathways. Core fucosylation has been shown to affect the binding between receptors and ligands, and further regulates the activity of downstream signaling pathways^45–47^. Therefore, we hypothesized that core fucosylation might be a promising “hub” target to prevent the progression of RIF and CKD.

Future research should be performed to evaluate the superiority of blocking multiple signaling pathways through core fucosylation over blockade of a single pathway in preventing RIF, to explore the underlying mechanisms regulating core fucosylation during the pericyte transition, and to clarify the precise locus of core fucosylation for the synthesis of small molecule drugs that modify glycosylation.

## Methods

All experiments in this study were performed at Key Laboratory of Liaoning Province and Renal Translational Medicine Center of Liaoning Province, China (2014225018).

### Collection of Samples from Patients with IgAN

Renal biopsy samples were obtained from the Department of Nephrology, The First Affiliated Hospital of Dalian Medical University. We selected 32 patients who were diagnosed with IgAN from January of 2015 to December of 2015. This study was approved by the Ethics Committee of The First Affiliated Hospital of Dalian Medical University (LCKY2013-14). All study methods were performed in accordance with the Declaration of Helsinki, and written informed consent was obtained from each subject prior to study initiation.

### Animal Care and UUO Mouse Models

C57BL/6 mice were obtained from the Dalian Medical University Laboratory Animal Center and housed at a constant room temperature with a 12 h light/dark cycle. Standard rodent chow and water were provided *ad libitum*. Animal experiments were conducted in accordance with the regulations established by the Institutional Committee for the Care and Use of Laboratory Animals and were approved by Dalian Medical University Laboratory Animal Center; all efforts were made to minimize suffering. All surgical procedures were conducted by a single surgeon under aseptic conditions in the Laboratory Animal Unit. Mice were anesthetized using an intraperitoneal injection of freshly prepared 10% chloral hydrate. A midline incision was made in the abdominal wall, and the left ureter was isolated and ligated using a 4.0 silk suture at two points along its length. The abdominal wound was closed with a silk suture, and mice were returned to their cages. Mice were humanely sacrificed on days 1, 3, and 7 (n = 6), and the kidneys were harvested for analysis.

### Histological Analysis of Kidney Tissues

IgAN or mouse specimens were fixed in 10% formaldehyde for 24 h. After dehydration, they were embedded in paraffin, and 1.5 μm thick cross-sections were stained with hematoxylin and eosin (HE), Masson’s trichrome, and periodic acid-Schiff (PAS). The sections were evaluated from five randomly selected fields by an independent pathologist (magnification of ×100). The extent of fibrosis was scored as 0 (negative), 1 (weak), 2 (medium), or 3 (intense). Each tissue section was observed under a light microscope (Olympus IX71, Tokyo, Japan) at magnifications of ×200 and ×400.

### Immunofluorescence Analysis of IgAN Renal Biopsies

A rotary paraffin microtome (Leica RM2255, Germany) was used to section the samples (1–2 μm). The sections were placed on polylysine-coated slides and incubated in a 60 °C oven. After dewaxing with xylene, rehydrating with 100 (5 min, twice), 95, 85, and 75% (5 min each) ethanol, and washing with PBS, the slides were placed in an EDTA antigen retrieval solution (0.01 M, pH 8.0). The slides were then placed in a microwave oven (850 W; Sharp, Osaka, Japan) until the antigen retrieval solution reached 100 °C for 10 min, cooled to room temperature for 20 min, and washed with PBS for 5 min. The slides were then placed in citrate antigen-repairing solution (0.01 M, pH 6.0) and heated in a high-pressure cooker until steam was observed. The slides were kept inside the cooker for 2 min, cooled to room temperature for 20 min, and washed with PBS for 5 min. Primary antibodies against PDGFRβ (Abcam, Cambridge, MA, USA), CD31 (Abcam, Cambridge, MA, USA), α-SMA (Abcam, Cambridge, MA, USA), and FUT8 (Santa Cruz Biotechnology, CA, USA) were incubated with the slides overnight at 4 °C, and LCA (Vector Labs) was incubated with the slides overnight at 4 °C. Secondary antibodies were incubated with the samples for 1 h at room temperature. Then, sections were mounted with Fluorescent Mounting Media containing 4′,6-diamidino-2-phenylindole (DAPI) (Abcam, Cambridge, MA, USA). Each tissue section was observed under a confocal laser scanning microscope (Leica SP8, Germany) at magnifications of ×400 and ×600, if necessary. Negative controls did not receive the first antibody.

### Immunofluorescence Staining of Mouse Kidney Tissues

Freshly harvested mouse kidney tissues were fixed with 4% paraformaldehyde (PFA) for 24 h. Then, tissues were successively dehydrated in 30%, 20%, and 10% sucrose solutions for 1 h each, and embedded in optimal cutting temperature compound (OCT, Tissue Tek, Sakura, Japan). Next, 4 μm cryosections were collected on Superfrost Plus glass slides. Sections were rinsed with PBS and permeabilized with a 1% Triton solution for 5 min. Then, they were blocked with blocking buffer (Vector Labs, CA, USA) for 1 h. Primary antibodies against PDGFRβ (Abcam, Cambridge, MA, USA), CD31 (Abcam, Cambridge, MA, USA), α-SMA (Abcam, Cambridge, MA, USA), and FUT8 (Santa Cruz Biotechnology, CA, USA) were incubated with sections overnight at 4 °C, and LCA (Vector Labs, CA, USA) was incubated with sections for 1 h at room temperature and then incubated overnight at 4 °C. Secondary antibodies were incubated with the samples for 1 h at room temperature. Then, sections were mounted with Fluorescent Mounting Media with DAPI (Abcam, Cambridge, MA, USA). Each tissue section was observed under a confocal laser scanning microscope (Leica SP8, Germany) at magnifications of ×400 and ×600, if necessary. Negative controls did not receive the first antibody.

### Pericyte Isolation and Pericyte-Myofibroblast Transition Model In Vitro

We used a previously described method^16^, with some modifications. Percoll was applied to remove impurities to reduce their impact on FACS analysis of pericytes. The kidney was diced and incubated with liberase (0.5 mg/ml, Roche, Mannheim, Germany) and DNase (100 U/ml, Roche) in Hank’s buffered salt solution for 45 min at 37 °C. After centrifugation, cells were resuspended in Hank’s buffered salt solution, and filtered (40 μm). Then, a 42% Percoll solution was used to remove impurities, and the cell layer was collected. Pericytes were purified by isolating PDGFR-β+ cells using FACS (FACSAria II, BD Biosciences) or MACS (Miltenyi, Germany), and were then cultured in pericyte medium supplemented with 2% FBS, 1% PGS, 1% P/S (ScienCell, CA, USA) at 37 °C in a 5% CO_2_ atmosphere with 90% humidity. The medium was changed every three days. Cells in the first or second passage were used for experiments. Recombinant TGF-β1 was used at a concentration of 5 ng/ml to induce the pericyte-myofibroblast transition.

### Immunofluorescence Staining of Pericytes

Cells were fixed with freshly prepared 4% paraformaldehyde for 10 min at room temperature. The cells were then washed three times with PBS. Each cover slip was then incubated in 1% BSA. Primary antibodies against PDGFRβ (Abcam, Cambridge, MA, USA), α-SMA (Abcam, Cambridge, MA, USA), or FUT8 (Santa Cruz Biotechnology, CA, USA) were incubated with the cells overnight at 4 °C, and LCA (Vector Labs, CA, USA) was incubated with the cells for 1 h at room temperature. After a wash with PBS, secondary antibodies conjugated with FITC or Cy3 were applied for 1 h at room temperature in a darkened humidified chamber. Finally, the preparations were washed with PBS, and mounted in fluorescent mounting medium with DAPI (Abcam, Cambridge, MA, USA). Images were acquired using a confocal laser scanning microscope (Leica SP8, Germany) at magnifications of ×200 and ×400. Negative controls did not receive the first antibody.

### Inhibition of FUT8 Expression

We constructed the adenovirus carrying the *FUT8* shRNA in our previous study^20^. The adenovirus carrying the *FUT8* shRNA was administered by tail vein injection of 1 × 10^6^ PFUs before anesthesia for UUO surgery. The *FUT8* siRNA was designed by a commercial company (RiboBio, 5′-GCUACUGAUGAUCCUACUUdTdT-3′) for FUT8 knockdown in pericytes *in vitro*. INTERFERin^TM^ (Polyplus-transfection SA, Bioparc, France) was used for transfection. Twenty-four hours after the start of transfection, pericytes were incubated with serum-free medium for 24 h. To confirm the knockdown of FUT8, we extracted RNA from cells and assessed FUT8 expression by reverse-transcriptase polymerase chain reaction (RT-PCR), according to the manufacturer’s protocol.

### Western Blot Analysis

Cells were collected and were lysed in RIPA buffer [1% Triton X-100, 150 mmol/L NaCl, 1 mmol/L EGTA, 50 mmol/L Tris-HCl, 0.1% sodium dodecyl sulfate (SDS), 1% sodium deoxycholate and phenylmethylsulfonyl fluoride (PMSF)] on ice for 20 min. Protein concentrations were determined with a BCA protein assay kit (Thermo, Rockford, IL, USA). Proteins (equal amounts, 10 μg/lane, were loaded) were separated on 6–10% SDS-PAGE gels and electrotransferred to polyvinylidene difluoride (PVDF) membranes (Bio-Rad, Hercules, CA, USA) at 400 mA for 2 h. Membranes were then incubated in blocking buffer (PBS with 0.05% Tween 20 and 0.1% BSA) for 1 h at room temperature, and then incubated with primary antibodies against α-SMA (Abcam, Cambridge, MA, USA), FUT8 (Abcam, Cambridge, MA, USA), TGFβR1 (Santa Cruz Biotechnology, CA, USA), PDGFRβ (Santa Cruz Biotechnology, CA, USA), Smad2/3 (Cell Signaling Technology, Beverly, MA, USA), ERK1/2 (Cell Signaling Technology, MA, USA), p-Smad2/3 (Cell Signaling Technology, MA, USA), and p- ERK1/2 (Cell Signaling Technology, MA, USA) overnight at 4 °C. Membranes were incubated with secondary antibodies for 1 h at room temperature. Blots were washed three times in a 1× PBS-Tween solution, and incubated with ECL reagents (Amersham, Pittsburgh, PA, USA) for 1 min. Proteins were visualized with super RX-N film (Fujifilm Corporation, Tokyo, Japan). A GAPDH antibody was used as a loading control to normalize the protein levels detected.

### Immunoprecipitation

Cell lysates were centrifuged (12,000 rpm for 20 min at 4 °C), and the supernatant was collected and precleared by Protein G PLUS-Agarose (Santa Cruz Biotechnology, CA, USA). Cell lysates (500 μg) were then incubated with 2 μg of anti-TGFβRII or anti-ALK5 antibody for 2 h at 4 °C on a rocker platform (30 rocks/min). Protein-antibody complexes were collected with 20 μl of Protein G PLUS-Agarose on a rocker platform (30 rocks/min) overnight at 4 °C. The immunoprecipitates were washed three times with lysis buffer. Equal amounts (10 μg/lane) of proteins were subjected to 12% SDS-PAGE for lectin blotting (described below). Negative controls did not receive the first antibody.

### Lectin Blotting

Immunoprecipitated TGFβR1 and PDGFRβ were separated on 12% SDS-PAGE gels and electrotransferred to PVDF membranes. The membranes were blocked with 5% BSA (wt/vol) in Tris-buffered saline containing 0.05% Tween 20 (TBST) overnight at 4 °C, and then incubated with TBST containing LCA-Biotin (Vector Labs, CA, USA), which preferentially recognizes Fuc-1,6GlcNAc, for 1 h at 23 °C. Blots were washed three times with a 1× PBS-Tween solution and incubated with ECL reagents (Amersham, Pittsburgh, PA, USA) for 1 min. The lectin-reactive proteins were visualized using super RX-N film (Fujifilm Corporation, Tokyo, Japan).

### Statistical Analysis

Results are presented as mean values ± standard deviations. Multiple comparisons of parametric data were performed using one-way analysis of variance (ANOVA). Nonparametric data were compared with the Mann-Whitney U-test to identify differences between groups. Pearson’s correlation analysis was calculated to determine the associations. A value of *P* < 0.05 was considered to indicate statistical significance. All statistical analyses were performed using the statistical package SPSS (version 21.0, IBM, Inc., USA).

## Electronic supplementary material

Supplemental file
