# Supplementary material for: Novel Mechanism of the Pericyte-Myofibroblast Transition in Renal Interstitial Fibrosis: Core Fucosylation Regulation
Source: Sci Rep. 2017 Dec 5;7:16914. doi: 10.1038/s41598-017-17193-5 (PMC5717002; doi:10.1038/s41598-017-17193-5)
Supplement: Supplementary file 1 — Supplemental file [file 41598_2017_17193_MOESM1_ESM.pdf]

Novel Mechanism of the Pericyte-Myofibroblast Transition in Renal Interstitial Fibrosis: Core Fucosylation Regulation

Nan Wang[1+], Yiyao Deng[1,5+], Anqi Liu[1,2], Nan Shen[1], Weidong Wang[1], Xiangning Du[1], Qingzhu Tang[1], Shuangxin Li[1,2], Zach Odeh[1,3], Taihua Wu [4*] and Hongli Lin[1*]

[+]***These authors contributed equally to this work.***

Corresponding author. *Hongli Lin: linhongli@vip.163.com. Or Taihua Wu: wutaihuadoc@126.com*

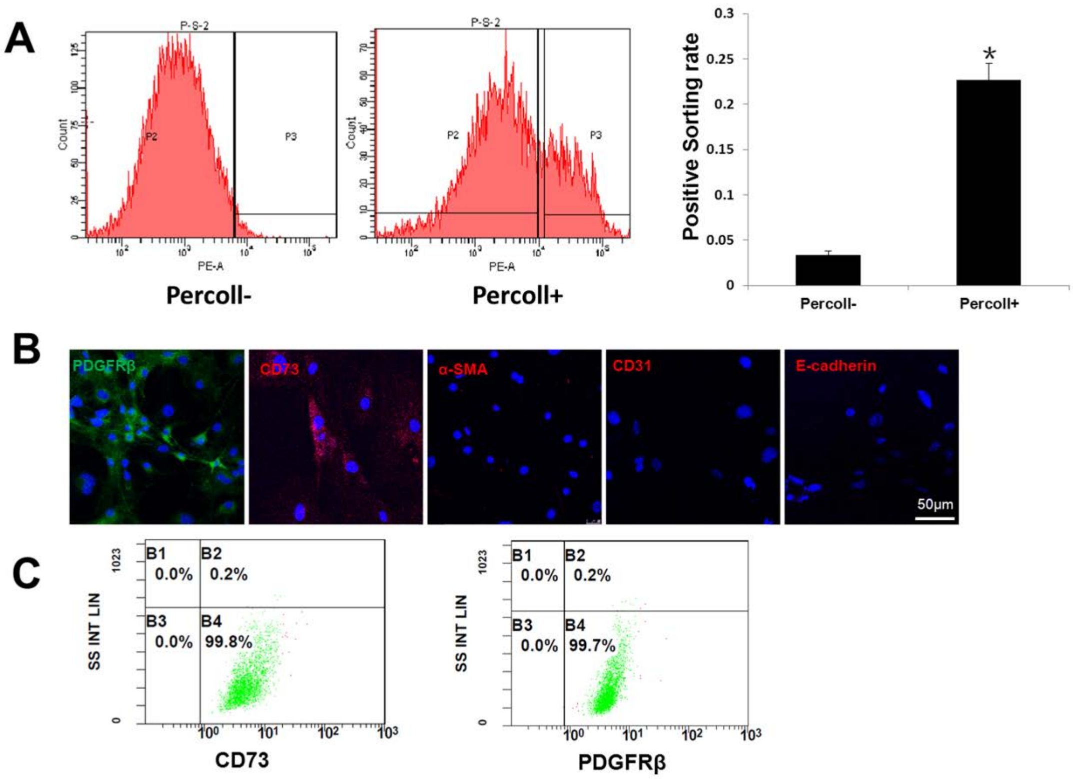

Figure S1. Percoll was used for pericyte isolation, and pericytes are identified. (A) The positive isolation rate was significantly increased when 42% Percoll was applied. (B) Sorted cells were PDGFR-β+ and CD73+, E-Cadherin-, CD31- and α-SMA-. (C) The purity of sorted cells was measured by flow cytometry, and the positive rate of CD73 and PDGFRβ was over 99%.

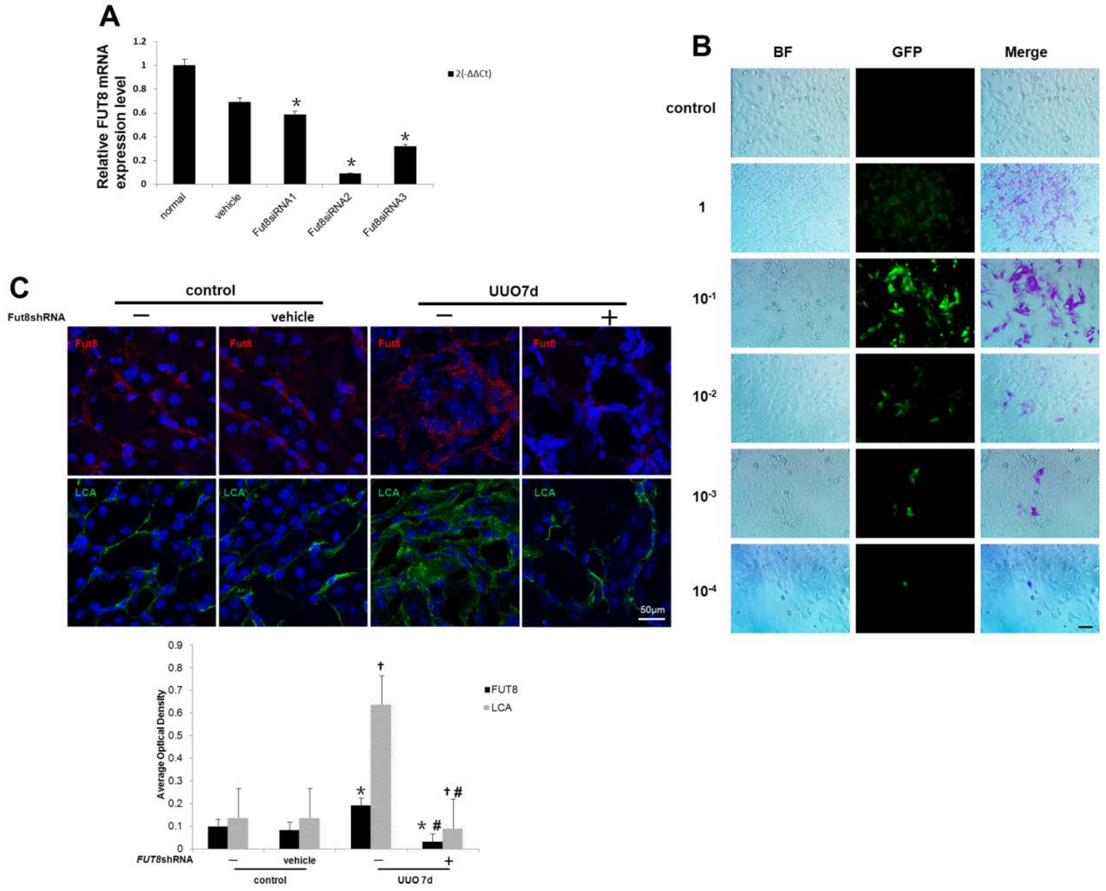

Figure S2. Validation of FUT8 knockdown efficiency. (A) *FUT8* siRNA was used for the knockdown of FUT8 in pericytes. (B) Titer of *FUT8* shRNA-adenovirus was determined in HK-2 cells. (C) FUT8 was decreased after the *FUT8* shRNA-adenovirus was applied. ***P<0.01, †P<0.01. ***** and † indicates control group versus UUO group; # indicates FUT8 knockdown group versus UUO group.
